# The Fetal Heart in Twin-to-Twin Transfusion Syndrome

**DOI:** 10.1155/2010/379792

**Published:** 2010-08-08

**Authors:** Tim Van Mieghem, Liesbeth Lewi, Léonardo Gucciardo, Philip DeKoninck, Dominique Van Schoubroeck, Roland Devlieger, Jan Deprest

**Affiliations:** University Hospitals Leuven, Division of Woman and Child, Department of Obstetrics and Gynecology, Fetal Diagnosis and Therapy Unit, Herestraat 49, 3000 Leuven, Belgium

## Abstract

Twin-to-twin transfusion syndrome is a severe complication occurring in 10% of monochorionic twin pregnancies. The disease is usually explained as due to an intrauterine imbalance in intertwin blood exchange, which leads to a volume depleted-donor twin and an overfilled recipient twin. The recipient has signs of cardiac dysfunction, which can be measured using echocardiography or blood and amniotic fluid derived biomarkers. Whereas cardiac dysfunction typically progresses in pregnancies treated with amniodrainage, it usually disappears within a few weeks after fetoscopic laser coagulation of the connecting intertwin anastomoses. Nevertheless, recipients remain at a increased risk of pulmonary stenosis. In this paper, we summarize the cardiac alterations in twin-to-twin transfusion syndrome, describe the changes seen after fetal therapy, list the newly proposed staging systems based on fetal cardiac function, and make recommendations about the use of fetal echocardiography in the evaluation and followup of pregnancies complicated by twin-to-twin transfusion syndrome.

## 1. Introduction

Monochorionic diamniotic twin pregnancies carry a 9%–15% risk of developing twin-to-twin transfusion syndrome (TTTS) [1, 2]. The pathophysiology of this disease is not fully understood, but the presence of vascular anastomoses connecting both fetal circulations at the level of the placenta is mandatory for its development. In carefully performed vascular injection studies, anastomoses have been documented in up to 95% of monochorionic placentas [3, 4], yet most of these pregnancies remain uncomplicated as the intertwin blood exchange is in balance. In a small subgroup however, the distribution of unidirectional arterial to venous anastomoses is imbalanced and an insufficient number of compensating bidirectional venovenous or arterio-arterial anastomoses is present, leading to a net shift of blood from one twin to the other [5, 6]. The pathophysiology and therapy of twin-to-twin transfusion syndrome have recently been covered in an extensive review [7].In brief, our current concept is that a net intertwin transfusion takes place over placental anastomoses, leading to volume shifts for which the fetuses cannot compensate. Additionally, plasma exchange and hormonal factors may play an important role in the disease. This ultimately results in a volume depleted donor twin, who will show signs of oligouria and oligohydramnios and a volume overloaded recipient twin who will present with polyuria and polyhydramnios. The diagnosis of TTTS is based on strict sonographic criteria reflecting severe intertwin fluid discordance. The criteria for TTTS are met when the deepest vertical amniotic fluid pocket is 2 cm or less in the donors amniotic sac. In Europe, gestational age-dependent criteria are used to define the polyhydramnios in the recipient twin (a deepest amniotic fluid pocket of more than 8 cm prior to 20 weeks and more than 10 cm after 20 weeks), whereas in the United States the 8 cm cutoff is used throughout gestation. The disease is currently staged based on the “Quintero system” which takes in account the filling of the bladder in the donor (stage I if the bladder is seen on ultrasound, stage II if not), the presence of arterial or venous Doppler flow abnormalities (stage III), the presence of fetal hydrops (stage IV) and intrauterine fetal demise (stage V) [8]. When left untreated, TTTS has a mortality and morbidity of up to 90%, mainly due to preterm rupture of the membranes and miscarriage or severe preterm birth as a result of the massive polyhydramnios [9]. However, intrauterine demise of one or both fetuses due to severe cardiac failure can also occur [10]. Treatment of severe midtrimester TTTS has shifted over the last 10 years from (repetitive) amniodrainage to fetoscopic laser coagulation of the connecting placental vessels. The latter therapy interrupts the intertwin transfusion and has been shown to improve neonatal survival and to decrease infant morbidity when compared to amniodrainage in a randomized trial [11, 12]. 

Although different research groups have focused on this disease and the number of publications on TTTS has risen exponentially over the last years, we still do not understand the exact nature of the disease [7]. Consequently, the currently used staging system does not describe the natural evolution of the disease, nor does it predict individual fetal survival after laser surgery adequately. Other insufficiently answered clinical questions are the prediction of the disease [13–16] and the optimal therapy for early (stage I) disease (expectant management, amniodrainage, laser) [17]. 

In an attempt to address the above questions, and with the advent of more sophisticated imaging tools in fetal cardiology [18], fetal medicine specialists and cardiologists have turned to comprehensive examination of the recipients heart. Indeed, one could expect volume shifts towards the recipient to be reflected in progressive cardiac failure. Consequently, a staging system based on the recipients cardiac function would make sense from a pathophysiological point of view and cardiac function assessment could theoretically be used for predicting the disease and for predicting recipient fetus survival after laser therapy.

This paper will update the reader on the fetal cardiac findings in TTTS and will discuss the indications of fetal echocardiography in TTTS.

## 2. Echocardiographic Findings in TTTS

### 2.1. Recipient Fetuses

Up to 70% of recipient fetuses of TTTS show some echocardiographic sign of cardiac compromise at the time of diagnosis [19], either at the anatomical or at the functional level. As such, in about half the cases, the heart is enlarged [20–22] due to an increased myocardial thickness [23] rather than to ventricular dilatation [10, 24]. In terms of systolic function, shortening fraction is considerably decreased in 30% of the recipients [10, 21, 22], and this predominantly at the level of the right ventricle [10]. Accordingly, speckle-tracking-derived measurements of strain and strain rate, although difficult to perform, show decreased strain in the right ventricle of recipient fetuses of TTTS [25]. In contrast to the lower contractility and to earlier reports that did not show differences in cardiac output between donors and recipients [22, 26], two recent series in relatively large cohorts of recipient fetuses have shown a moderate increase in cardiac output when corrections were made for fetal weight [23, 27]. This finding clearly fits in with the volume overload theory.

In TTTS, diastolic function is even more compromised than systolic function. As a consequence of the thickened, dysfunctional myocardium, monophasic ventricular filling patterns such as those seen in restrictive cardiomyopathy occur in about 20%–30% of cases, again with a predominance on the right side [21, 28]. Moreover, we often observe a shortening of the ventricular filling time [29], a prolongation of the isovolumetric relaxation time [30] and an increase in the Tei-index (which is a geometry independent indicator of both systolic and diastolic function based on the assessment of the isovolumetric relaxation and the isovolumetric contraction time [31, 32]). On average, the Tei-index is 40% higher than normal [23, 30, 33] and values above the upper limit of normal are observed in about 50% of cases [24, 28, 30]. Interpretation of the Tei-index in the fetal setting nevertheless deserves particular caution as fetal blood pressure is often unknown and prolongation of the isovolumetric contraction time can be a reflection of hypertension rather than of systolic dysfunction. Therefore, separate analysis of the isovolumetric contraction and relaxation time is justified, yet only technically possible at the level of the left ventricle due to the implantation of the pulmonary and tricuspid valve precluding simultaneous recording of the pulmonary and tricuspid flow.

Tricuspid regurgitation occurs in about 30%–50% of recipients [21, 28, 34] but is severe in only half of these [10, 21–23]. Mitral regurgitation on the other hand is much less frequent (6%–14% of cases) [21, 28], yet usually severe (9%) [21]. The presence of valvular regurgitation allows to estimate fetal blood pressure using the Bernouilli equation and studies have shown that recipient fetuses display marked hypertension with systolic pressures over 2-fold the normal value for gestational age [35]. 

Further down the vascular tree, Doppler assessment of the ductus venosus and the umbilical venous flow allows to estimate the right atrial pressure curve. Reversed flow in the ductus venosus and umbilical vein pulsations have been integrated in the Quintero staging system and their presence upstages the disease to stage III. In most series from tertiary referral centers, abnormal ductus venosus dopplers are seen in about 1 in 3 recipients [21, 23, 28, 34] and a pulsatile umbilical vein in 1 in 10 [21, 22, 28].

A summary of the fetal echo findings in a prospective series of 78 consecutive cases seen in our unit is presented in Table 1 (unpublished data). It is important to note that in Quintero stage I, already 45% of cases show signs of ventricular dysfunction in terms of an increased Tei index and that 35% of cases have a fused right ventricular inflow pattern suggestive of diastolic dysfunction. The occurrence of these so-called early findings remains relatively stable over stage I to III, similar to what has been published earlier [24].

Nevertheless, other findings such as the left ventricular Tei-index and mitral and tricuspid regurgitation increase with Quintero stage [21] suggesting that the Quintero staging system, at least to some degree, reflects progressive fetal cardiovascular compromise.

Our group has shown that changes in cardiac function are already present well before the actual development of TTTS. As such, about 30% of fetuses with moderate amniotic fluid discordance not fulfilling the criteria of TTTS but ultimately progressing to the syndrome show an increased myocardial performance index [36]. Along the same line, 40% of monochorionic twins that ultimately will develop TTTS have already abnormal findings in the ductus venosus flow [1, 13] or discordant nuchal translucency measurements reflective of altered hemodynamics in the first trimester of pregnancy [14, 15, 37]. Unfortunately, these findings are not very specific, nor very sensitive. They cannot therefore be used for early prediction of the disease, nor should they be used to “upstage” (often benign) fluid discordance to TTTS. 

**Figure 1:**
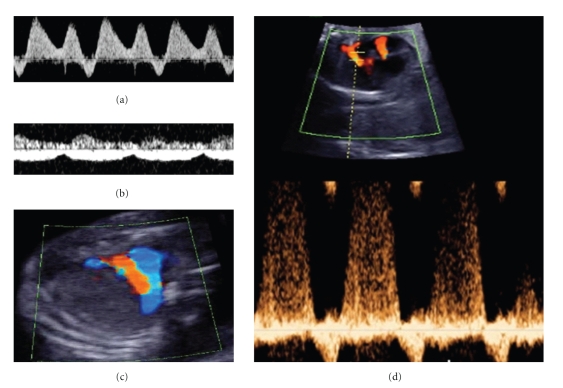
Common echocardiographic findings in the recipient of TTTS. (a) Reversed flow in the ductus venosus. (b) Umbilical vein pulsations. (c) Transverse view of the fetal chest at the level of the 3-vessel view demonstrating forward flow in the aorta (blue) and reversed flow in the ductus arteriosus and pulmonary artery (red) suggestive of functional pulmonary atresia. (d) Doppler assessment at the level of the fetal 4-chamber view demonstrating mitral and tricuspid regurgitation with the corresponding pulsed Doppler spectrum below.

Once a TTTS is fully installed, echocardiographic findings tend to progress over time, with worsening ventricular hypertrophy and systolic dysfunction, which can ultimately lead to fetal hydrops and intrauterine fetal demise [38]. Moreover, as growth of fetal cardiac structures is dependent on the blood flow through them, persistent ventricular dysfunction can lead to secondary anatomic changes. Consequently, in a consecutive series of 150 recipient fetuses, 16% had a smaller than expected right ventricular outflow tract at the time of initial presentation [21]. In up to 4%, extreme right ventricular dysfunction can result in functional pulmonary atresia (Figure 1) with retrograde perfusion of the pulmonary trunk through the ductus arteriosus [10, 34] and more rarely even in complete right heart flow reversal [39].

### 2.2. Donor Fetuses

In contrast to recipient fetuses, donors seem to have a normal cardiac function, yet some 5%–10% present with abnormal Doppler waveforms in the ductus venosus, and 3% with tricuspid regurgitation or umbilical vein pulsations [34, 40], findings which are generally explained by the presence of severe placental insufficiency. The latter is also supported by an increased occurrence of abnormal diastolic flow in the umbilical artery in the donor fetus. 

Furthermore, although not significant in most studies, the donor twin has a trend towards a lower Tei-index than in the normal population which is suggestive of hypotension [27, 40]. Finally, there have been speculations about an increased incidence of aortic coarctation in donors due to a lower venous return from the placenta and hence a decreased loading of the left ventricular outflow tract [41].

## 3. Biomarkers of Altered Fetal Hemodynamics in TTTS

Different vasoactive peptides have been investigated in TTTS, mainly in an attempt to further explain the underlying pathophysiological mechanisms.

The renin angiotensin aldosterone system has been found to be upregulated in the donor kidney [42]. Transfer of these hormones towards the recipient through the placental anastomoses partly explains the hypertension (angiotensin II) and the hypervolemia (aldosterone) seen in this fetus. Additional upregulation of atrial natriuretic factor (ANF) has been observed in recipients when compared to donor fetuses [43]. Plasma levels of ANF are correlated with the amount of amniotic fluid yet not with the severity of cardiac dysfunction [43] and are therefore thought to mediate the recipients polyuria.

Increased endothelin-1 [44], brain or b-type natriuretic peptide (BNP) [28, 44, 45] and cardiac troponin T [28] levels have been observed in the plasma and/or the amniotic fluid of recipient fetuses, similar to observations in adults with chronic heart failure. Endothelin-1 can certainly play a role in the development of the severe hypertension [35, 44], stimulates the myocardial remodeling [46] and could decrease cardiac function. The presence of both BNP and cardiac troponin T [28] suggests that the myocardium is not only stretched by the volume load but also that it undergoes structural damage/remodeling.

## 4. New Staging Systems in TTTS

In an attempt to provide a more pathophysiologic classification of TTTS [47], different groups have suggested to use new staging systems that are mainly based on the severity of cardiac dysfunction in the recipient fetus. The most extensive system has been elaborated by the Children's Hospital Of Philadelphia (CHOP) [21] and requires the evaluation of 12 variables which, in experienced hands, takes 30–45 minutes per fetus and is therefore not feasible in routine clinical practice (Table 2). Also, different parameters of cardiac function are correlated. For example, we have shown that the ejection fraction correlates with the myocardial performance index [33] and others demonstrated that abnormal flow in the ductus venosus correlates with tricuspid regurgitation [34]. Finally, Rychik et al. [21] showed that the right ventricular Tei-index was strongly correlated with their full 12 parameter score, suggesting that the creation of an easier staging system, still encompassing the full extent of the disease should be feasible.

Going further into this, Stirnemann et al. [23] used cluster analysis and partitioning algorithms to determine that a staging system including only the assessment of the left and right ventricular myocardial performance index allows to stratify cases as well as a system with additional inclusion of shortening fractions, ductus venosus pulsatility index and cardiac output. A comparison of the anatomic and functional parameters in the different proposed “cardiac” staging systems is presented in Table 2.

At present, we do not feel that these new staging systems should replace the Quintero system, which is an easy and widely accepted method for patient stratification that has proven some usefulness in terms of predicting fetal outcome after laser therapy [11, 49]. Nevertheless, cardiac staging systems may play an important role in the further understanding of the pathophysiology of the disease and are useful in research settings.

## 5. Effect of Prenatal Therapy

Amniodrainage usually does not cure TTTS but is rather a palliative and repetitive intervention aimed at relieving the polyhydramnios. As such, it does not improve fetal cardiac function and fetuses undergoing repetitive amniodrainage show progressive cardiac disease and hydrops and are at risk for intrauterine demise [10, 50].

On the other hand, closure of the vascular anastomoses at the level of the placenta and functional separation of both fetuses by fetoscopic laser leads to a rapid improvement in cardiac function in the recipient fetus. Already in the first 48 hours after therapy, cardiac size, precordial venous Dopplers, valvular regurgitation, and ventricular inflow patterns normalize in about half of the cases and the Tei-index improves with approximately 40% [19, 20, 40, 51]. Survival is worse in fetuses lacking this functional improvement immediately after surgery [19]. In the longer term, further amelioration in cardiac function continues and approximately 6 weeks after therapy most cases have regained normal cardiac function [40]. The normalization of cardiac dysfunction is very similar to, but slightly faster than, what is seen in neonates delivered at the time of TTTS [52]. Interestingly, even severe cardiac dysfunction such as functional pulmonary atresia and hydrops resolve in almost all cases [51, 53], which argues against the use of selective reduction in these fetuses.

In contrast to recipients, about 1 in 4 donor fetuses has a temporary worsening in cardiac function with increased cardiac size [20], tricuspid regurgitation, ductus venosus alterations, and subcutaneous oedema [34, 40, 54] after fetoscopic laser therapy. These findings however disappear by 2–4 weeks after the surgery [40, 54] and are probably explained by the sudden arrest of the transfusion imbalance and temporary relative volume overload in the former donor fetus.

Different groups have investigated whether fetal demise after laser therapy (which occurs in about 18% of recipient fetuses [49]) can be predicted by preoperative fetal cardiac function. In a retrospective series, Shah and colleagues showed that recipient cardiovascular profile score can predict outcome to a certain extent [48]. In line with this finding, we have shown that recipient fetuses with a normal Tei-index and low amniotic fluid cardiac troponin T levels have an improved survival compared to those with alterations in either of these 2 parameters. However, cardiac function alone does not predict outcome [28], as confirmed in a larger multicenter series including more than 200 TTTS cases [55]. This is explained by the fact that fetal demise after laser is multifactorial and also depends on other factors such as placental sharing or incomplete laser separation. For clinical practice, it means that for now, fetal therapy cannot be tailored to the individual situation based on fetal cardiac function assessment.

## 6. Long Term Cardiac Outcome after TTTS

Followup until the age of 10 year has shown that both donors and recipients of nonlasered TTTS have normal cardiac function in the longer term [56]. Recipient twins nevertheless maintain a slightly reduced early diastolic ventricular filling as compared to donors (diastolic dysfunction). Donors on the other hand seem to have higher arterial wall stiffness than recipients, suggestive of intrauterine vascular programming [57]. Fetoscopic laser therapy can alter this prenatal vascular programming. As such, fetuses that underwent laser have normal wall stiffness and normal cardiac function at the age of 2 year [58, 59]. However, the increased occurrence of right ventricular outflow tract obstruction observed at the time of TTTS (16%) [21] does not disappear completely and recipient fetuses remain at a 3-fold increased risk (5%–8%) of pulmonary stenosis at the time of birth when compared to uncomplicated monochorionic twins [22, 59].

## 7. Clinical Recommendations

In clinical practice, the main question remains whether (functional) fetal echocardiography should be used in the evaluation and follow-up of pregnancies complicated with TTTS and if the answer is yes, when echocardiography should be performed. From the above listed data, we feel that the only clinically useful echocardiographic finding in TTTS pregnancies booked for fetoscopic laser therapy is the presence of persistent pulmonary artery stenosis after therapy which would impact on the place of delivery and on postnatal management. As a result, we would recommend a thorough (structural) cardiac evaluation 8–10 weeks after the fetoscopic surgery, when cardiac dysfunction has completely resolved, to assess pulmonary artery development and to plan the site of delivery.

In TTTS pregnancies managed expectantly or undergoing (repetitive) amniodrainage, the evidence is less clear, yet we feel they should undergo intensive cardiac follow-up with at least assessment of ductus venosus and umbilical vein flow and evaluation for the presence of hydrops to time eventual delivery or to switch therapy to laser before intrauterine fetal demise occurs. Additionally, these fetuses should undergo evaluation for pulmonary artery stenosis before birth. 

As all recipient fetuses of TTTS, both managed conservatively or with laser, are at increased risk of pulmonary artery stenosis, we are convinced that an early postnatal screening echocardiogram is indicated. Moreover, because all monochorionic twins are at increased risk for structural cardiac abnormalities compared to singletons or dichorionic twins [22], all should benefit from midtrimester structural echocardiographic assessment.

## 8. Conclusions and Future Perspectives

In summary, cardiac dysfunction is a common finding in recipient fetuses and different new “cardiac” staging systems have been proposed. Although they may bring new pathophysiologic insights, their clinical value remains limited as they do not predict the occurrence nor the outcome of the disease. However, further evaluation is necessary in stage I disease, where equipoise is still present about the optimal treatment strategy [17]. Additionally, the impact of the decreased cardiac function on cerebral perfusion and long-term neurologic development requires further investigation. Fetoscopic laser coagulation of the vascular anastomoses interrupts the intertwin transfusion and has been shown to lead to fast normalization of cardiac function. Nevertheless, recipients remain at increased risk of pulmonary artery stenosis. Further work should be directed at detecting prenatally which twins will have clinically important lesions at the time of birth.

## Figures and Tables

**Table 1:** Occurence of cardiac function alterations in 78 consecutive recipient fetuses assessed at the University Hospitals Leuven, Belgium.

	Stage I (*n* = 11)	Stage II (*n* = 19)	Stage III (*n* = 42)	Stage IV (*n* = 6)	Overall (*n* = 78)
Reversed a-wave ductus venosus (%)	0	0	62	50	37
Umbilical vein pulsations (%)	0	0	50	67	32
Fusion of RV inflow (%)	36	37	38	83	41
Tricuspid regurgitation (%)	9	21	38	67	31
RV-MPI > percentile 97.5 (%)	45	47	48	83	49
Fusion of LV inflow (%)	9	21	29	67	27
Mitral regurgitation (%)	0	0	10	50	10
LV-MPI > percentile 97.5 (%)	25	42	38	83	41

RV: right ventricle, LV: left ventricle, and MPI: myocardial performance index.

**Table 2:** Comparison of the cardiac parameters assessed in the different proposed staging systems.

	Quintero et al. 1999 [8]	Rychik et al. 2007 [21]	Shah et al. 2008 [48]	Habli et al. 2008 [19]	Stirnemann et al. 2010 [23]
		“CHOP-score”	“Cardiovascular profile score”	“Cincinatti staging”	
Cardiothoracic ratio		x	x		
Ventricular wall thickness		x		x	
Shortening fraction		x	x		
Tei-index right ventricle				x	x
Tei-index left ventricle				x	x
AV regurgitation		x	x	x	
AV inflow		x			
Pulmonary insufficiency		x			
Outflow tract size		x			
Ductus venosus	x	x	x	x	
Umbilical vein	x	x	x	x	
Hydrops	x		x	x	
Umbilical artery donor	x	x	x	x	

AV: ventricular valve.
